# A Case of Chronic Wernicke Encephalopathy (WE): An Underdiagnosed Phenomena

**DOI:** 10.7759/cureus.19100

**Published:** 2021-10-28

**Authors:** Hina Bagash, Assad Marwat, Asghar Marwat, Bruce Kraus

**Affiliations:** 1 Internal Medicine, Rawalpindi Medical University, Rawalpindi, PAK; 2 Internal Medicine, Shifa Tameer-E-Millat University, Shifa College of Medicine, Islamabad, PAK; 3 Internal Medicine, Prairie Ridge Health, Sun Prairie, USA; 4 Internal Medicine, Conemaugh Memorial Medical Center, Johnstown, USA; 5 Internal Medicine, Prairie Ridge Health, Columbus, USA

**Keywords:** thiamine deficiency, korsakoff psychosis, parenteral thiamine, chronic alcoholism, wernicke encephalopathy

## Abstract

Wernicke encephalopathy (WE) is the most common neurological complication of thiamine deficiency in patients who have a background of chronic alcohol use disorder. WE is characterized by acute onset of confusion, gait ataxia, and oculomotor dysfunction. Prompt treatment with parenteral thiamine leads to improvement. Untreated WE has mortality rates of up to 20% and many cases progress to the more chronic Korsakoff syndrome. Cases of untreated WE in which symptoms last beyond the acute phase and become chronic are rarely found in the literature. Here, we present a case of a 64-year-old female having a background of chronic alcohol use disorder presenting with symptoms of gait ataxia, recurrent falls, and decreased concentration. These symptoms had progressed over a period of nine months. The patient was seen by her family physician and several specialists undergoing many diagnostic studies with inconclusive results. Ultimately, with a high index of suspicion for thiamine deficiency, she was admitted for IV thiamine treatment. Upon follow-up in the clinic, the patient reported improvement in her balance and concentration further confirming the initial suspicion of WE with thiamine deficiency as the cause of her symptoms. This case corroborates the existence of WE as a chronic phenomenon in addition to the more commonly reported acute WE. Furthermore, this case highlights the importance of recognizing WE as a potential cause of chronic neurological symptoms in people with alcohol-related disorders and the role of IV thiamine in treatment.

## Introduction

The principal neurological consequence of thiamine deficiency in the setting of chronic alcohol use disorder is Wernicke-Korsakoff syndrome [1-2]. Wernicke encephalopathy (WE) was first described by Carl Wernicke in 1881 as an acute encephalopathy, and a few years later, Sergei Korskoff delineated a chronic amnestic syndrome affecting memory out of proportion to other cognitive deficits. Thus, Wernicke-Korsakoff syndrome chronicles different stages of the same disease process [3].

WE is an acute neurological condition characterized by a clinical triad of ophthalmoparesis with nystagmus, ataxia, and confusion [1,4-5]. All features of the classic triad are present in only approximately one-third of patients; in most, only one or two elements of the clinical triad are apparent. The absence of one or more of the classic symptoms likely leads to underdiagnosis [1,5].

Treatment of WE requires prompt administration of parenteral thiamine, which, if given in a timely manner, is a life-saving measure that may also avert the development of chronic brain damage [6]. WE is associated with considerable mortality and morbidity with fatality rates of up to 20% in patients that are either untreated or inadequately treated [7-9]. The remaining patients develop varying levels of brain damage, although the specific course of the disease is not well-established [10].

Approximately 80% of people with an alcohol-related disorder recovering from classic WE exhibit selective memory disturbance of Korsakoff Psychosis (KP), whereas a small minority will have minor or no cognitive deficits [11]. KP is a syndrome defined by chronic amnesia, learning deficits, and short-term memory loss, with improvement in the confusion caused by the initial acute WE within one to two weeks [11-14]. Cases of untreated WE in which symptoms last beyond the acute phase and become chronic are rarely found in the literature [15]. Here we present a case study of a 64-year-old female with signs and symptoms of WE that were untreated and became chronic progressing over a year. The patient was finally treated with IV thiamine resulting in partial improvement.

## Case presentation

We present a 64-year-old female with a past medical history of generalized anxiety disorder, major depression, post-traumatic stress disorder, hypertension, gastroesophageal reflux disease, neurogenic bladder, and alcohol use disorder who presented to her primary care provider with symptoms of loss of balance with recurrent falls, memory loss with mild confusion, and visual changes, including impaired depth perception and loss of peripheral vision. These symptoms were ongoing for the past several months. The patient reportedly had one to two falls per week. The patient worked as a registered nurse in a psychiatric facility and was found to be unsteady with cognitive dysfunction at work. Subsequently, the patient was asked to take medical leave for evaluation before being able to return to work. Initially, the patient did not report significant alcohol use and denied any illicit drug use.

**Figure 1:**
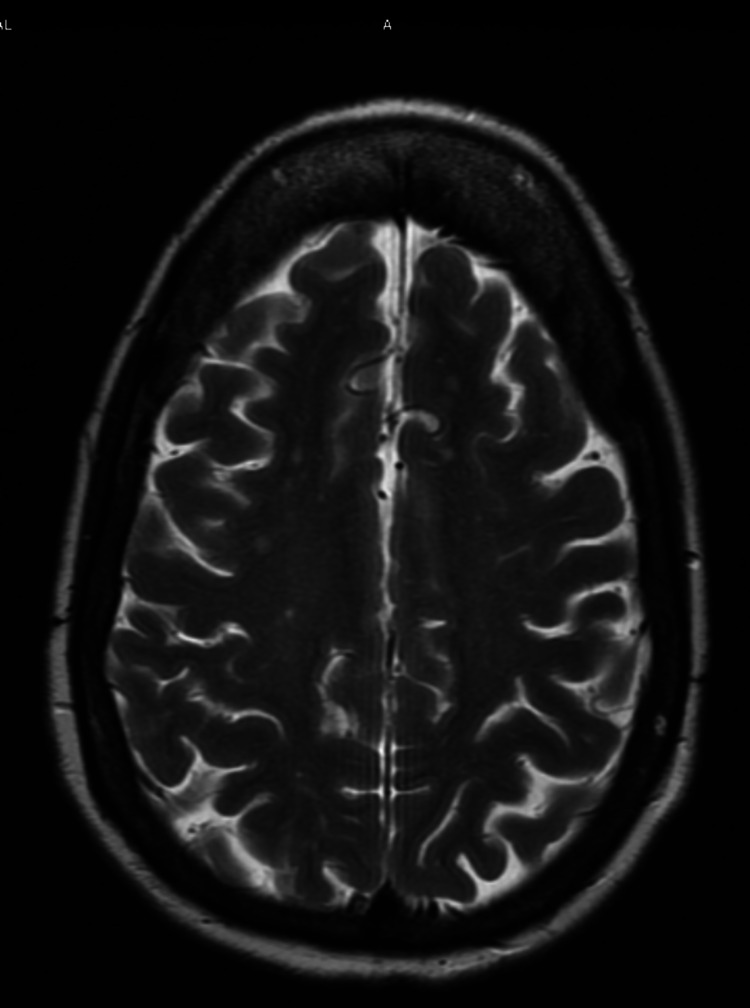
MRI brain showing microangiopathic changes

The initial general physical examination was unremarkable. On neurological exam, the only pertinent finding was a wide-base ataxic gait. Initial laboratory studies showed a WBC count of 5.7*103/ul, hemoglobin of 13 gm/dl, platelets of 234*103/ul, sodium of 139 mmol/l, potassium of 4.1 mmol/L, creatinine of 1.29 mg/dl, thyroid-stimulating hormone (TSH) of 1.78 uIU/ml, urinalysis (UA) was unremarkable, blood alcohol level <10 mg/dl, vitamin B1 level of 122 nmol/L, vitamin B12 of 630 pg/ml, and folate of 11.3 ng/ml. The patient also underwent an MRI of the brain with and without contrast showing chronic microangiopathic change and stable mild generalized parenchymal loss with no evidence of acute intracranial pathology or mass (Figure 1).

The patient was subsequently referred for specialist consultation with neurology and neuro-ophthalmology. She then underwent neuropsychometric testing, the results of which were within the normal limits. The patient also underwent a detailed ophthalmic exam and electrophysiological testing for her visual symptoms, which also did not provide a clear explanation of her symptoms. Ultimately, the patient reported to her primary care provider on follow-up visits that her alcohol intake was much higher than what she had previously reported. She reported that she started drinking heavily around four years ago following her father’s death due to difficulty coping. She was drinking three to four glasses of nine ounces of wine on a daily basis.

At this point, the patient’s symptoms had been ongoing for close to a year. WE was postulated as a possible cause of her ongoing neurological symptoms in the light of her recently reported excess alcohol use and no alternate diagnosis available despite an exhaustive diagnostic workup. It was decided to treat the patient with intravenous thiamine. The patient was admitted and treated with IV thiamine 250 mg three times a day for two days and then once daily for an additional five days.

Upon outpatient follow-up, the patient noted some improvement in her symptoms. She self-reported much less confusion and improvement in her balance clinically confirming the diagnosis of WE. The patient continued to receive oral thiamine; however, despite initial rapid improvement in her symptoms, her progress plateaued. She continued to have some residual neurological deficits, including problems with balance and mild confusion although much less pronounced than before.

## Discussion

Thiamine is a vital part of several enzymatic and metabolic pathways in the central nervous system [13,16]. Only a few weeks of dietary deficiency of thiamine is enough to deplete the body stores [9]. Falling levels of thiamine cause thiamine-dependent enzyme systems associated with the prevention and recovery of cellular damage in the CNS to be impaired. This damage to thiamine-dependent metabolic pathways leads to brain lesions commonly seen in WE and KP. Thiamine deficiency and WE are common in malnourished patients, particularly in those with chronic alcohol-related disorders [14].

The classic triad of altered mental status, ataxia, and ocular signs although clinically indicative of WE are neither diagnostic nor pathognomonic for the disease. Furthermore, the absence of these symptoms does not rule out WE. Typical MRI findings of reversible cytotoxic edema typified by symmetric alterations in the thalami, mammillary bodies, tectal plate, and periaqueductal area may not be present in every case, and some patients may have no MRI findings [9,17-18].

Due to the nonspecific and poorly recognized nature of WE, we must maintain a high index of suspicion and empirically treat high-risk patients [14]. Although a large body of research currently exists, which is dedicated toward the successful management of the life-threatening acute phase of WE, case reports describing prolonged confusion are currently lacking. This is particularly compelling as in clinical practice despite established standards of emergency medicine in the industrialized world WE is still underdiagnosed and a considerable number of patients with WE are sub-optimally treated. This lack of treatment results in patients with prolonged confusional states [19-21].

The current case study under discussion demonstrates that WE can not only present as an acute life-threatening condition but can also manifest as a more chronic form of chronic cognitive disturbance with balance and gait problems requiring lifelong care. This chronic disease process can place a significant burden on our healthcare delivery systems. Furthermore, our patient showed some improvement with parenteral thiamine therapy but did not achieve complete resolution of symptoms. This partial recovery shows that the prognosis of WE depends on timely diagnosis and early treatment with thiamine.

## Conclusions

This case illustrates that the chronic sequela of untreated WE is still not completely understood in some patients and further study into this phenomenon is the need of the hour. The key to avoiding chronic neurological symptoms in these patients is recognizing and treating WE early on. This case also provides us with a valuable lesson that IV thiamine is not only beneficial in acute WE but also in patients presenting with chronic symptoms. It is also striking that WE continues to be undertreated in acute settings, resulting in chronic neurological symptoms although this has been rarely studied. Our case study highlights the importance of recognizing thiamine deficiency as a cause of neurological symptoms even if the presentation is not acute. Parenteral thiamine is an effective way to treat patients with symptoms of balance and altered mental status in people with chronic alcohol-related disorders who do not have another explanation of their symptoms.
